# Comparison of Two Surgical Techniques for the Treatment of Canine Disc Associated-Cervical Spondylomyelopathy

**DOI:** 10.3389/fvets.2022.880018

**Published:** 2022-06-20

**Authors:** Cristian Falzone, Vito Tranquillo, Nicola Gasparinetti

**Affiliations:** ^1^Department of Neurology-Neurosurgery, Diagnostica Piccoli Animali, Zugliano, Italy; ^2^Istituto Zooprofilattico Sperimentale della Lombardia e dell'Emilia Romagna, Bergamo, Italy

**Keywords:** cervical spondylomyelopathy (CSM), Wobbler syndrome, prosthetic disc, distraction stabilization, surgical procedures

## Abstract

**Objective:**

To compare prosthetic disc and vertebral distraction stabilization in dogs with disc-associated cervical spondylomyelopathy (DA-CSM).

**Study Design:**

A retrospective clinical study.

**Animals:**

25 dogs.

**Methods:**

Dogs presenting with clinical signs and MRI findings compatible with DA-CSM underwent surgery. Implantation of the Adamo's prosthetic disc (PD) or vertebral distraction-stabilization (DS) with intervertebral cage, ventral locking plates, and dorsal transarticular screws was performed. All dogs were followed-up and evaluated clinically for a minimum of 1 year and radiographically for at least 3 months. In particular, we focused on the evaluation of subsidence (the degree of vertebral collapse).

**Results:**

Twenty-five dogs were enrolled: 12 with PD implantation and 13 with DS implantation. Of these, 24 dogs were followed-up at 1 year. Overall, 12 dogs improved (4 PD and 8 DS), eight were stable (4 PD and 4 DS), and four deteriorated (3 PD and 1 DS). Deterioration was more common in PD cases, especially soon after surgery. In a few PD cases, a second surgery was necessary. The most common complication in dogs with DS was discospondylitis. Subsidence was detected in 11 PD and 7 DS dogs. Subsidence was more severe and occurred sooner after surgery in PD cases compared to DS cases. DS cases were more prone to clinical improvement and less prone to subsidence than PD cases in this study. However, the statistical evidence was weak owing to the small sample size.

**Conclusion:**

The preliminary results suggest that prosthetic disc implantation is more prone to clinical and radiographic failures than distraction stabilization.

**Clinical Relevance:**

The DS technique is a valuable surgical option for treating dogs with DA-CSM, with favorable short- and long-term clinical and radiographic outcomes.

## Introduction

Canine disc-associated cervical spondylomyelopathy (DA-CSM), also known as caudal cervical spondylomyelopathy (CCSM) or disc-associated Wobbler syndrome (DAWS), affects particularly Dobermann Pinschers, and also other breeds of large dogs (1–6). Typical neurological signs include “two engines gait,” with ataxia of the pelvic limbs and hypometria of the thoracic limbs (1, 2). DA-CSM is usually a progressive disease. Due to cervical spinal cord compression and secondary damage, caused by degenerative disc disease and protrusion, hypertrophy of the dorsal longitudinal, and sometimes, the interarcuate ligaments is evident (1, 4, 7, 8). Most cord compressions in DA-CSM cases tend to have a dynamic component, which fluctuates the cord compression, making it sometimes worse and sometimes alleviated, for example, in extended or traction positions (9–15). Although magnetic resonance imaging (MRI) is commonly accepted as the method of choice for establishing a diagnosis, controversy still exists on how to treat this condition.

Surgical treatment offers more benefits than medical treatment (1, 11, 16–26). A variety of surgical techniques have been proposed for DA-CSM, with many of the authors claiming success rates between 70 and 90% (27–44). Surgical options include the distraction-stabilization of the affected vertebral segments and more recently, the implantation of a prosthetic disc (PD) (11, 32–49). The goal of both surgeries is to relieve spinal cord compression. However, the prosthetic disc aims to achieve relatively normal vertebral motion, whereas distraction-stabilization aims for vertebral fusion (29–44, 47–49). In this study, we retrospectively analyzed the records of dogs with DA-CSM treated via prosthetic disc implantation or vertebral distraction-fixation and evaluated the clinical outcomes and the imaging findings. Radiographs and magnetic resonance images, at different times after surgery, were evaluated. We also determined whether subsidence had occurred on follow-up radiographs.

## Materials and Methods

### Animals

Dogs presented to the Diagnostica Piccoli Animali between January 2014 and September 2018 with clinical signs and MRI findings compatible with DA-CSM and treated by surgery were included. Dogs were included if they demonstrated a typical clinical presentation of the “two engines gait,” with ataxia of the pelvic limbs and hypometria of the thoracic limbs. The study and animal rights were reviewed and approved by the dedicated internal institutional Diagnostica Piccoli Animali committee. The dogs were divided into two groups, representing the two surgical techniques. An approximately equal number of dogs with similar body weight, age, severity, and duration of neurological signs were included in each group.

One criterium for selecting dogs for PD treatment was the complete resolution of spinal cord compression on MRI after traction. This was based on previous observations by the authors (unpublished), that dogs with residual cord compression on MRI and PD implantation had a worse recovery compared to dogs with complete traction-responsive lesions. Neurological status was compared pre- and post-operatively, and dogs were rated as deteriorated, stable, improved, or normal. Outcomes were evaluated within 30 days (short-term follow-up), at over 30 days and within 1 year (medium-term follow-up), and at over 1 year or more (long-term follow-up).

### Pre-operative Diagnostics

The definitive diagnosis of DA-CSM was confirmed by radiographic and MRI studies. Imaging was performed using either a low-field (Vet Grande-Esaote, 0.27T) or a high-field unit (Achieva-Philips, 1.5T), in neutral and post-traction positions, as previously described (49). The definitive MRI diagnosis was formulated based on the presence of disc protrusion with secondary cord compression and damage, in association with various degrees of abnormalities of the vertebral bodies and joints, in conjunction with the typical clinical presentation as stated above.

### Surgical Treatment

Each dog was premedicated with intravenous (IV) or intramuscular (IM) methadone 0.3 mg/kg (Semfortan®, Eurovet Animal Health B.V). General anesthesia was induced with IV fentanyl 2 μg/kg (Fentadone®, Eurovet Animal Health B.V) and propofol (Proposure®, Boehringer Ingelheim Animal Health Italia S.p.A.) and titrated to effect with a total dose of 4–6 mg/kg. The dogs were then intubated and maintained with oxygen and isoflurane (Isoflo®, Zoetis Italia S. r. l.; MAC 1.3%) or sevoflurane (Sevoflo®, Ecuphar Italia S. r. l., MAC 2.3%). Analgesia was provided using a CRI of fentanyl, 8–10 μgr/kg/h. Cephazolin sodium (22 mg/kg; Cefazolina®, Teva Italia S. r. l.) was administered intravenously after anesthetic induction and was repeated every 120 min until the surgical procedure was complete.

Dogs were placed in dorsal recumbency for the ventral slot. The decision to make a full or partial ventral slot in the treated spaces depended on the presence or absence of residual cord compression on the post-traction MRI images. Before performing the ventral slot, a self-retaining Caspar distractor was used to distract the affected spaces. A full slot was used in dogs with residual cord compression after traction to access the vertebral canal and remove the protruded disc until the spinal cord was visible. A partial slot was used in dogs with completely resolved cord compression after traction to access and thin the inner layer of the annulus, without entering the vertebral canal. Adamo's prosthetic disc (PD) implantation or distraction-stabilization (DS) followed the ventral slot. DS was performed using an intervertebral cage (Cervlock®, PorteVet, size 1: length 15 mm and width 7 mm) and two ventral titanium locking plates of 2.7 mm (PAX Locking System®, Securos). The cages were titanium and filled with freeze-dried and reconstituted bovine bone (Bio-Oss®, Geistlich biomaterials). Bone graft was also placed above the cage itself. After routine closure, DS cases were placed in sternal recumbency and a routine dorsal approach was performed to the affected vertebral articulation. Transarticular fixation was added by placing a titanium screw across each articular facet (2.7 mm or 3.5 mm according to the size of the facet). The facets were covered with the bovine bone graft for the DS procedure (40, 49).

During the first 24–36 h post-operatively, each dog received IV cefazolin twice daily, IM methadone every 4–6 h, and IM carprofen 2 mg/kg (Rimadyl®, Zoetis Italia S. r. l.). Each dog was discharged with a 5-day course of oral cephalexin 20 mg/kg (ICF Vet®, Industria Chimica Fine S.r.l.), carprofen twice daily, and tramadol 2 mg/kg three times daily (Tralieve®, Le Vet Beheer B.V.).

### Post-operative Imaging

**Figure 1:**
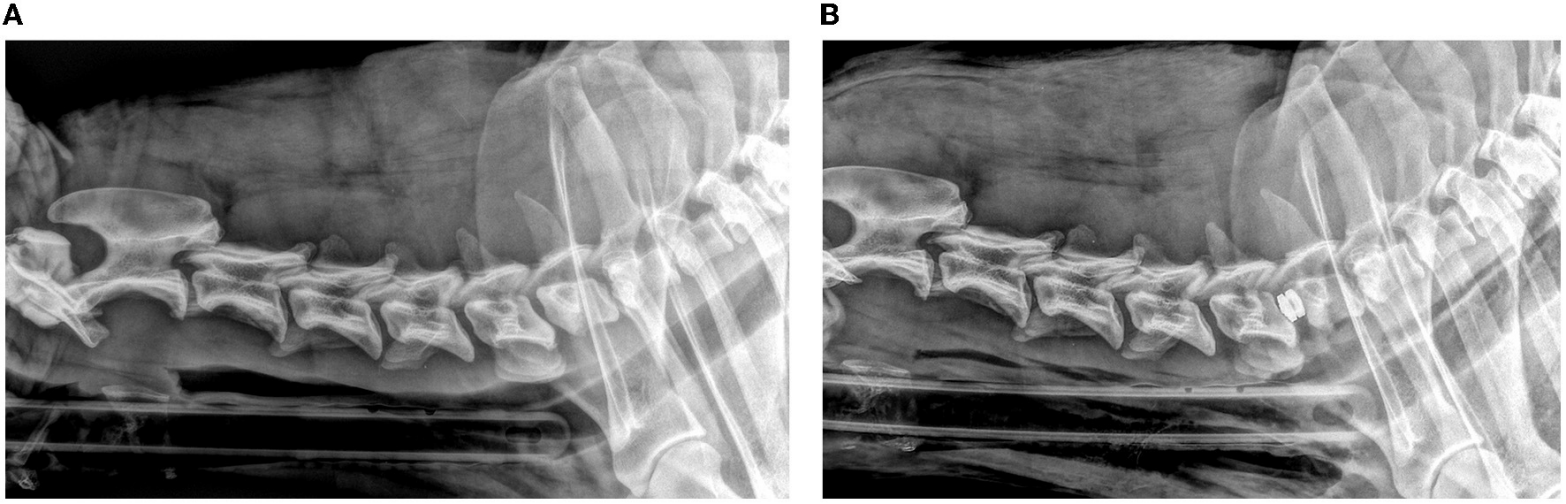
Lateral pre-operative **(A)** and post-operative **(B)** radiographic views of a dog with prosthetic disc implantation.

**Figure 2:**
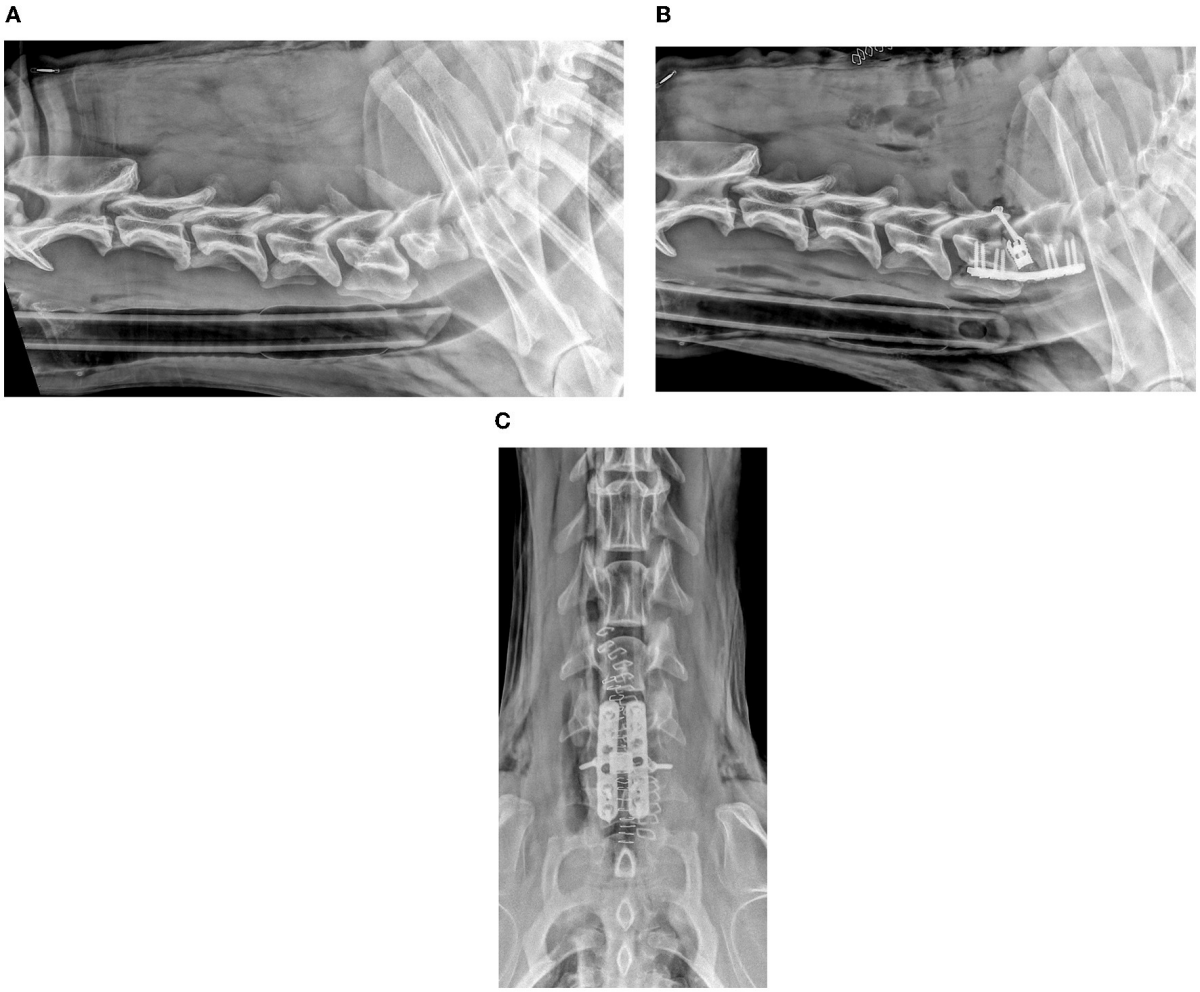
Lateral pre-operative **(A)** and post-operative **(B)** and ventro-dorsal post-operative **(C)** radiographic views of a dog with distraction-stabilization using intervertebral cage, two parallel ventral locking plates, and two dorsal trans-articular screws.

Radiographs were taken immediately post-operatively, and at 1 and 3 months after surgery (Figures 1, 2) for evaluation. Radiographic evaluation mostly focused on subsidence, described as the percentage of distraction loss between the dorsal aspect of the adjacent treated vertebral endplates. Two authors separately measured the distance between the two dorsal aspects with open-source software for navigating multidimensional DICOM images (Osirix, www.osirixviewer.com) at the time of surgery and follow-ups. Distraction loss between immediate post-operative and follow-up radiographs was calculated as a percentage. Subsidence was rated as mild (10 and 25% distraction loss), moderate (25–50%), severe (more than 50%), and none (0–10%, as a minimum human margin of error was considered possible and acceptable). The degree of vertebral fusion/bone production among the dogs treated with DS and the degree of residual motion among the dogs treated with PD were also considered using dynamic X-rays (neutral, flexed, and the dorsal extended position of the cervical column). Other changes, such as breaking of a screw or signs suggestive of discospondylitis were also noted. MRI images were repeated at different times after surgery. Implant position, residual cord compression at the treated sites, new sites of cord compression, vertebral collapse or changes, such as bone lysis, and intra-medullary changes, such as parenchymal hyperintensity on T2 weighted images, were evaluated. Furthermore, parenchymal hypointensity on T1 weighted images was reported.

### Statistical Analyses

Statistical analysis was performed using a generalized linear mixed model (GLMM) to compare the subsidence grade of the different treatment options (PD vs. DS), with dogs considered as random effects. Intraclass-coefficient-correlation (ICC) was calculated from the variance components of the model to assess the agreement between the authors on subsidence degree (49).

Outcomes after surgery were considered as a multinomial non-ordered categorical variable with three levels: “improved,” “stable,” and “worse.” A multinomial logistic regression [MLR; Venables WN, Ripley BD (2002)] was fitted to predict the outcomes using treatment modality as a predictor factor. All data analyses were performed using R programming language and environment for statistical computing (R Core Team, 2020), with lme4 for GLMM and nnet for MLR. Accessory packages such as readxl, tidyverse, sjPlot, and ggeffects were used to handle the data and make the table and the graphs.

There is a growing consensus within the biomedical scientific community not to use the significant/non-significant dichotomy based on a predetermined *p*-value cut-off for result interpretation. Recently, the American Statistical Association (ASA) has also cautioned the use and meaning of the *p*-value. As such, we focused on the extent of the estimated treatment effect and its uncertainty. We reported the *p*-value exclusively as a measure of the evidence against the null hypothesis (the treatment is equal and therefore the degree of subsidence between the two treatments is 0), without establishing a cut-off *p*-value to define significance (50–54).

## Results

### Demographics

In total, 25 dogs fulfilled the inclusion criteria. The Dobermann pinscher was the most represented breed (13/25). There were also four Weimaraner, two Bernese Mountain dogs, and one each of the following: Rottweiler, Deutsch Kurzhaar, Hannoverscher Schweisshund, German Shepherd, Beauceron, and Mongrel (Table 1). The mean age was 8.7 years (range, 4–10 years). There were 14 males and 11 females. All dogs were ambulatory at presentation, with typical ataxia, mild to moderate paresis of the hind limbs, and hypometria or floating gait of the thoracic limbs. The mean duration of the presenting clinical signs before surgery was 94 days (minimum 10 days, maximum 1 year), with acute and subacute onset, in 3 and 22 of 25 cases, respectively.

**Table 1:** Dog's signalment, intervertebral disc space affected, and the surgical technique applied, PD (prosthetic disc) vs. DS (distraction-Stabilization); Degree of subsidence is also illustrated: none (between 0 and 10%), mild (below 25%), moderate (between 25 and 50%), and severe (more than 50%).

**Patient**	**Disc affected and surgical technique**	**Subsidence**
Dobermann, Male 8 year old	PD, C6-C7	Moderate
Dobermann, Female, 6 year old	PD, C6-C7	Mild
Weimaraner, Male, 7 year old	PD, C6-C7	Mild
Hannoverscher Schweisshund, male, 4 year old	PD, C6-C7	Severe
Bernese Mountain dog, Male, 5 year old	PD, C6-C7	Mild
German Shepherd dog, Male, 7 Year old	PD, C6-C7	Severe
Weimaraner, Female, 6 year old	PD, C5-C6, and C6-C7	Moderate
Bernese Mountain dog, Female, 6 year old	PD, C6-C7	Moderate
Dobermann, Male, 8 year old	PD, C5-C6, and C6-C7	Lost follow-up
Dobermann, Female, 8 year old	PD, C6-C7	Severe
Dobermann, Female, 8 year old	PD, C6-C7	Severe
Rottweiler, Male, 8 year old	PD, C6-C7	Moderate
Baeuceron, Male, 10 year old	DS, C6-C7	Moderate
Dobermann, Male, 6 year old	DS, C6-C7	Moderate
Dobermann, Female, 6 year old	DS, C6-C7	None
Dobermann, Female, 6 year old	DS, C6-C7	Lost follow-up
Mongrel, Female, 10 year old	DS, C6-C7	None
Deutsch Kurzhaar, Male, 8 year old	DS, C6-C7	None
Dobermann, Female, 7 year old	DS, C6-C7	Moderate
Dobermann, Male, 8 year old	DS, C6-C7	Moderate
Weimaraner, Male, 7 year old	DS, C5-C6, and C6-C7	None
Weimaraner, Male, 7 year old	DS, C6-C7	Mild
Dobermann, Male, 8 year old	DS, C5-C6, and C6-C7	Mild
Dobermann, Male, 7 year old	DS, C6-C7	None
Dobermann, Male, 7 year old	DS, C6-C7	None
Hannoverscher Schweisshund, male, 4 year old	DS, C6-C7	Mild

### Imaging Diagnosis

Based on the inclusion criteria, a definitive diagnosis of DA-CSM was confirmed via radiographic and MRI findings in all dogs. In 21 cases, only the C6-C7 site was affected. In the remaining four dogs, both C5-C6 and C6-C7 were involved. The affected intervertebral disc spaces were reduced in all cases. Vertebral body abnormalities were detected in all the Doberman and Weimaraner dogs, but not in any other dogs. On MRI, intramedullary hyperintensity on T2 weighted images was observed in the spinal cord parenchyma over the affected spaces in all cases, varying from mild to severe, and becoming more evident in terms of size and signal intensity with chronicity. In 18/25 dogs, the cord compression dramatically improved after traction.

### Intraoperative Findings

Twelve dogs were treated with PD implantation (2 cases: C5-C6 and C6-C7 and 10 cases: C6-C7) and 13 with DS (2 cases: C5-C6 and C6-C7, 11 cases: C6-C7). The mean surgery duration in the PD group was 1 h 18 min for dogs with C6-C7 disease and 2 h for those with a double space. In the DS group, the mean duration was 3 h for a single space and 4 h for two sites. Despite being rare, the most common intraoperative complication was bleeding from the venous sinuses. This occurred in four dogs that had full ventral slots from the DS group.

### Clinical Outcomes

**Figure 3:**
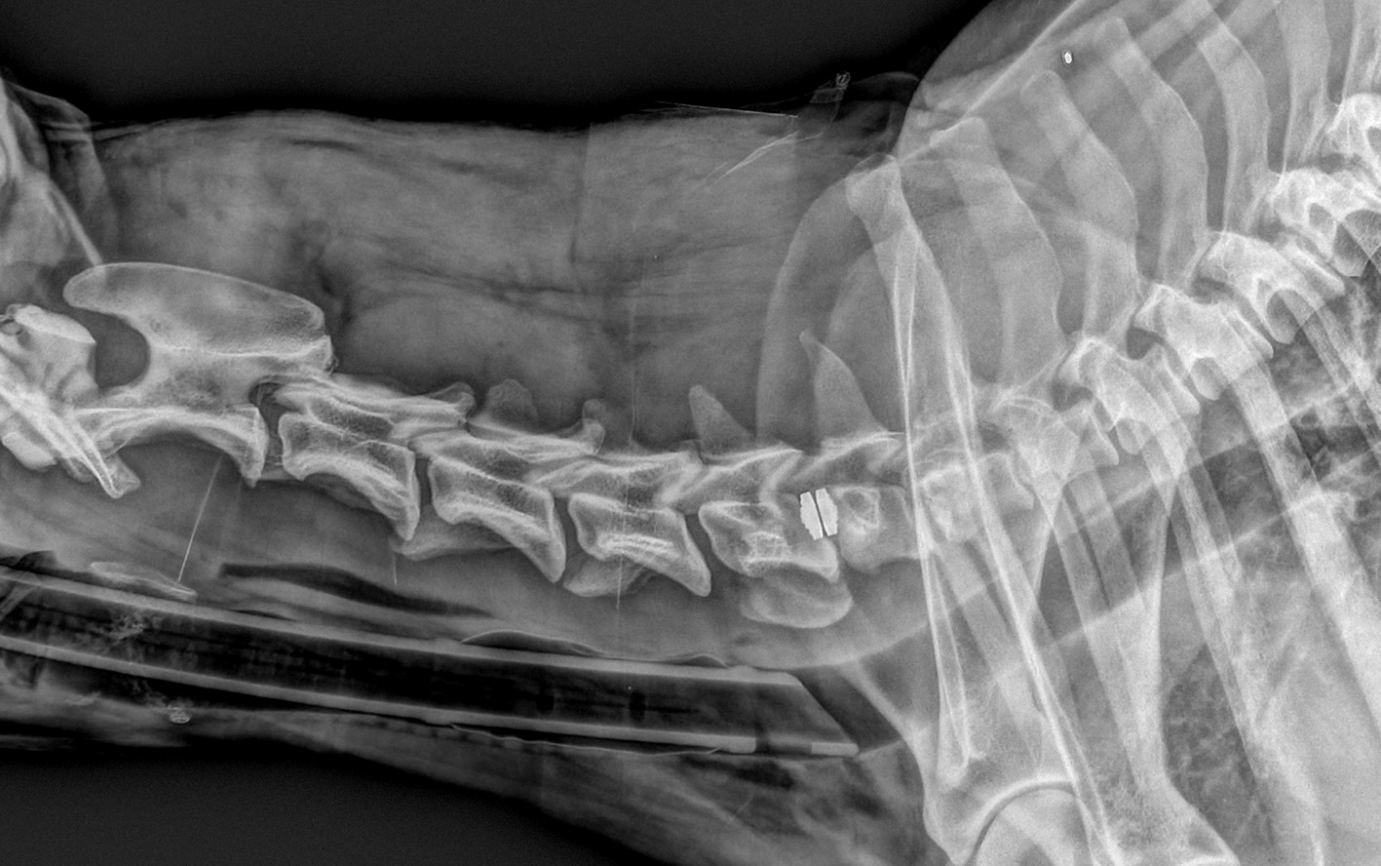
Same dog as in Figure 1, with severe neurological deterioration and subsidence at 9 days after surgery.

Follow-up at different time intervals are summarized in Table 2. All dogs were discharged within 24–36 h after the surgery. Very mild deterioration, presenting as a subtle worsening of the pre-existing ataxia or paresis, was observed in five dogs that had full ventral slots, and they all improved within a week. Within the 1st month after surgery (short-term follow-up), three dogs deteriorated, and all were from the PD group. One of these dogs developed a minimal ambulatory tetraparesis at 9 days due to severe subsidence and relapse of the cord compression, as evident in the repeat radiographs and MRI (Figure 3). Two dogs developed severe neck pain and mild to moderate tetraparesis, at 15 and 25 days, respectively. The tetraparesis was due to residual nucleus pulposus extrusion in the previously treated spaces associated with moderate subsidence in both cases, as shown in the repeat MRI. These three dogs required a second surgery. One was treated with the DS technique and the other two dogs required removal of the extruded disc through a lateral approach (55, 56).

**Table 2:** Number of dogs and their post-operative outcome (stable, improved, and deteriorated) at different follow-up times (short-term: within 30 days; medium-term: over 30 days and within 1 year; long-term: over 1 year), for both surgical technique (PD, prosthetic disc; DS, Distraction-Stabilization).

**Clinical outcome**	**Short term (25 dogs: 11PD, 14DS)**	Medium term (24 dogs: 11PD, 13DS)^*^	Long term (24 dogs: 11PD, 14DS)^*^
Stable	3 PD - 3 DS	6 PD - 2 DS	4 PD - 4 DS
Improved	5 PD - 11 DS	3 PD - 8 DS	4 PD - 8 DS
Deteriorated	3 PD	2 PD - 3 DS	3 PD - 1 DS

* *Twenty four dogs were available for medium- and long-term follow-up, for a sudden death of one DS dog*.

Among the remaining 22 dogs, 16 improved (5 PD and 11 DS) and 6 were stable (3 PD and 3 DS).

Twenty-four dogs were available for clinical re-evaluation between 1 month and 1 year after surgery (medium-term follow-up). One dog from the DS group suddenly died 30 days after surgery due to unknown reasons and, therefore, was not followed-up. Among those 24 dogs, 11 improved (3 PD and 8 DS), eight cases were stable (6 PD and 2 DS), and five dogs were worse than their pre-operative status (2 PD and 3 DS). The three dogs that deteriorated from the DS group were diagnosed with discospondylitis at 45 days, 6 months, and 10 months after surgery, respectively. In these three dogs, discospondylitis was initially suspected on radiography and then confirmed on MRI. It affected the intervertebral disc space cranial to the treatment site in two dogs and the space caudal to the treatment site in one case. These three dogs received oral amoxicillin and clavulanic acid (20 mg/kg BID, Synulox®, Zoetis Italia S. r. l.) and enrofloxacin (5 mg/kg SID, Baytril®, Elanco Italia S. p.A.), for a duration of 90–120 days. Both dogs deteriorated from the PD group developed neck pain at 2 and 4 months after surgery and one also became mildly tetraparetic. Radiographically, both dogs showed severe subsidence and were treated conservatively with a tapering dose of prednisolone and gabapentin (10 mg/kg BID). The neck pain improved in both dogs with a mean time of 60 days, but the gait did not recover. All three dogs with PD that deteriorated within the 1st month of surgery showed improvement after the second surgery. The dog was previously treated with PD was reoperated with DS-technique at 9 days because of severe subsidence with cord compression relapse and was clinically monitored and radiographically assessed for more than 1 year, although it was not included in the statistical analysis.

At 12 months after surgery (long-term follow-up), 24 dogs were available for neurological assessment. The results were compared to their pre-operative status. Of the 24 dogs, 12 improved (4 PD and 8 DS), eight were stable (4 PD and 4 DS), and four deteriorated (3 PD and 1 DS). The DS dog deteriorated at 2 years and became more ataxic on the pelvic limbs; this dog underwent an MRI scan 3 years post-operative, which showed progressive intramedullary damage, as evident by a more severe parenchymal hyperintensity on T2 WI. As mentioned, all three dogs from the DS group that developed discospondylitis improved. Two dogs in the PD group that deteriorated at the medium-term follow-up, improved pain-wise, but one remained tetraparetic and was included in the group of the three PD-dogs that deteriorated. The cause for deterioration was not investigated in the remaining two PD-dogs. Owners of 15 dogs were available for telephone follow-up at 2 years post-operative; they reported an unchanged status from the last follow-up (5 PD and 10 DS). Six dogs were available for follow-up at 3 years (2 PD and 4 DS), including the DS-dog that deteriorated because of the progression of the intramedullary damage, as showed by the repeat MRI. Five dogs remained the same as their last assessment.

Statistically, at the short-term follow-up, dogs treated with PD were 6.60 times more likely to be stable than dogs treated with DS, 8.80 times more likely than dogs treated with DS to be worse, and an odds of 0.11 lower than DS dogs to be improved. At the medium-term follow-up, PD dogs were 8.00 times more likely to be stable and 2.67 times more likely to be worse than DS dogs. PD dogs also had an odds ratio of 0.38 lower than DS dogs to be improved. At the long-term follow-up, PD dogs had an odds ratio of 2.50 times greater than DS dogs to be stable, an odds of 6.00 times greater than DS dogs to be worse, and an odds ratio of 0.17 lower than DS dogs to be improved.

Overall, the statistical analysis suggested a higher chance to deteriorate or being stable using the PD technique, whereas with the DS technique, dogs were more likely to improve. However, the 95% CI was very large for all categories examined, and therefore statistical results must be interpreted with caution (**Figure 7**).

### Radiographic Outcomes

**Figure 4:**
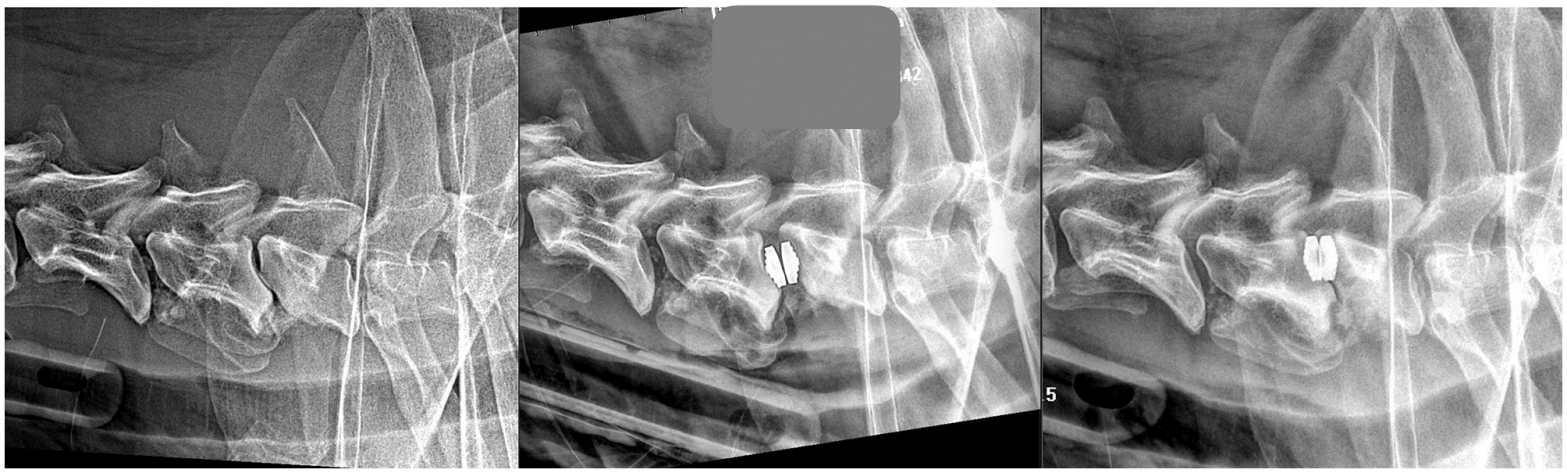
Latero-lateral radiographs of the same dog before a PD surgery (left image), at 30 days post-op (middle image) and at 90 days post-op (right image).

All the dogs were radiographed immediately post-operative. Radiographs were repeated in 23 dogs, from 30 to 90 days. Four of these 23 dogs also had extra radiographs at 6 months and 2, after 1 year. Two dogs were lost to medium-term radiographic follow-up: the DS-dog that had died unexpectedly and one PD-dog where the owner declined imaging. Of the 23 dogs available for radiographic evaluation, subsidence was identified in 11/12 dogs in the PD group and 7/13 dogs in the DS group. In the 11 dogs in the PD group, subsidence was more evident over time and varied from mild in four patients, to moderate in three dogs, and severe in four cases (Table 1). As previously stated, the two dogs with disc extrusions had moderate subsidence. The dog in the PD group that deteriorated after 9 days had severe subsidence: this dog was reoperated by the DS technique. The remaining three cases with severe subsidence had significant new bone production in the treated sites 90 days after radiographic reassessment. In one dog, almost complete vertebral fusion was detected (Figure 4). In contrast, in two dogs, a mild residual vertebral movement was evident on the flexion and extension radiographs. Of these three dogs, one was neurologically unchanged compared to their pre-operative assessment, while two deteriorated clinically, with neck pain in both cases and mild tetraparesis in one dog. These dogs were treated conservatively, with partial improvement. In the PD group, the degree of vertebral motion evaluated in the flexed and extended neck position was preserved in only one dog with mild subsidence, while it decreased to absent in the remaining dogs in proportion to the severity of subsidence. A high tendency for vertebral fusion with new bone production and spondylosis was radiographically more evident in cases of moderate and severe subsidence.

**Figure 5:**
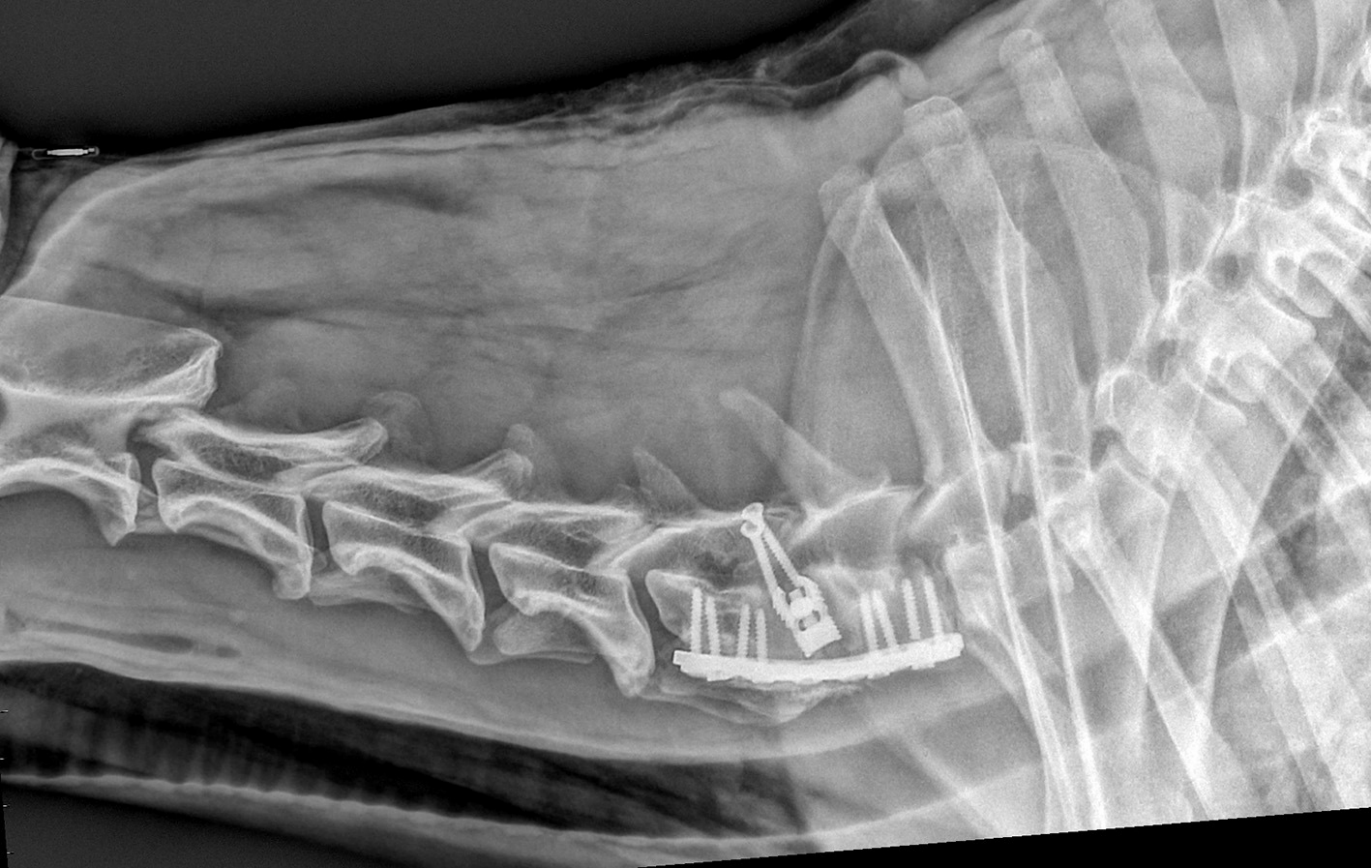
Same dog as in Figure 2, with one screw rupture at 90 days radiographic recheck.

In the seven dogs in the DS group, subsidence was rated as mild in three cases and moderate in four. No dogs suffered from severe subsidence (Table 1). Subsidence was associated with the breaking of a screw in three dogs (two screws in two cases and one screw in one case, Figure 5) and with discospondylitis in the dog that deteriorated 45 days after surgery. No major radiographic implant failure or other significant changes were noticed in the remaining three cases. Six dogs had no subsidence at 3 months. Of these six dogs, five also showed no subsidence when X-rays were repeated later, at 6, 10, 12, 16, and 36 months. Good bone production with vertebral fusion was observed in all dogs. This observation was more obvious radiographically at and after 90 days.

The mean subsidence was 47.62 mm in the PD group and 18.59 mm in the DS group; a relevant reduction in subsidence of 29.03 mm (39%) was observed in dogs treated with DS (95% CI: −47.78 −10.28), as observed from the GLMM model results, with strong evidence against the null hypothesis that the surgical techniques are equal in terms of subsidence grade (*p* = 0.02). A very high level of agreement was observed between the two authors in rating the degree of subsidence (ICC = 0.97).

**Figure 6:**
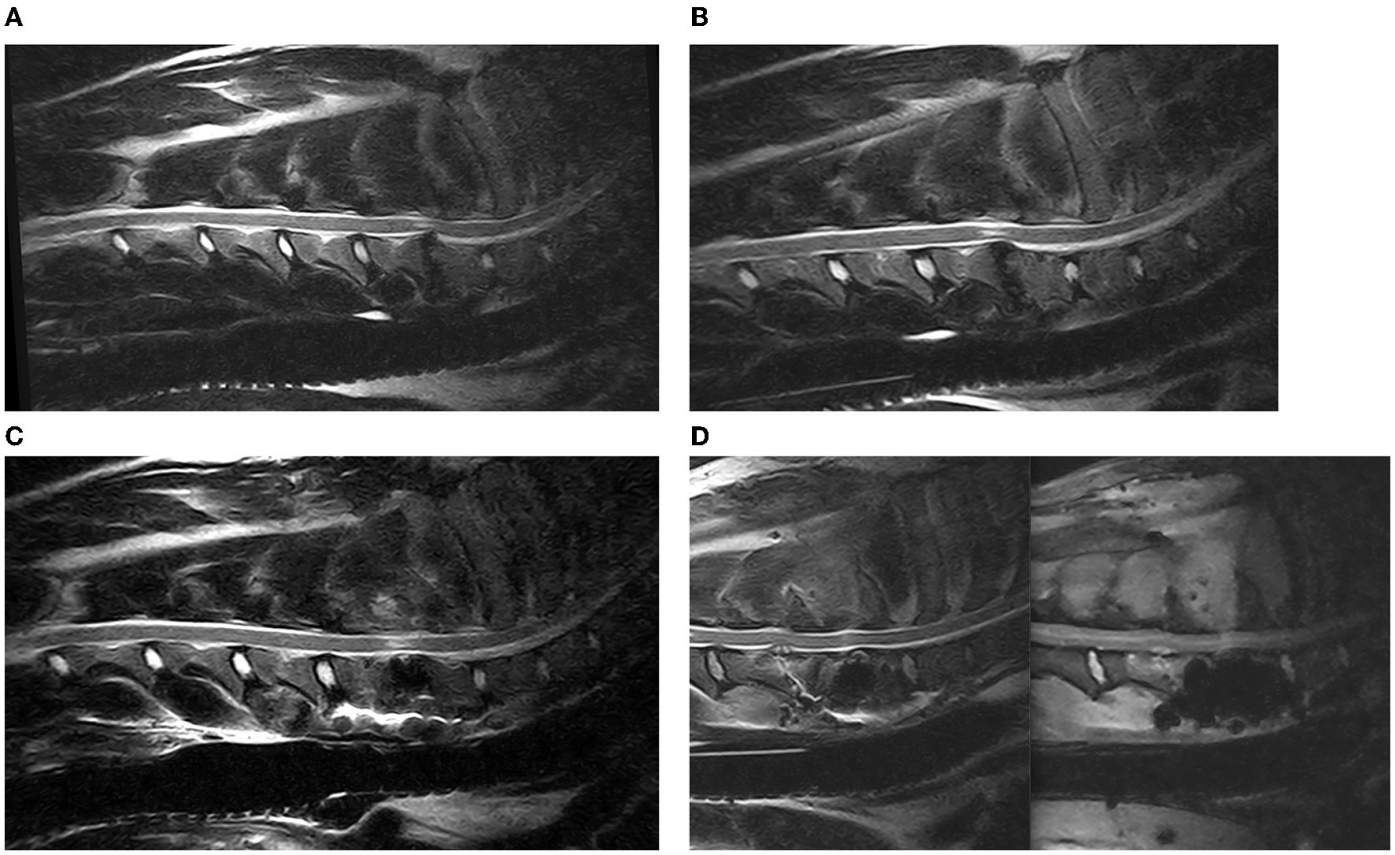
Sagittal T2 weighted images of a DA-CSM-dog, pre- **(A)** and post-traction **(B)** and 3 months post DS-surgery **(C)**; from left to right sagittal T2 Weighted and STIR Images of an example of discospondylitis at C5–C6, in a patient previously treated by DS at C6–C7 **(D)**.

MRI was repeated in 14 dogs (7 PD and 7 DS) for a total of 18 studies. Seven were acquired immediately after surgery (two PD dogs and five DS dogs) and showed good implant positioning with none to minimal residual cord compression in all cases. Six cases were imaged due to neurological worsening: three PD-dogs at 9, 15, and 25 days (disc extrusion and moderate subsidence in two cases and severe subsidence with relapse or cord compression in one case) and three DS cases, respectively, at 45 days, 5 months, and 10 months, all with discospondylitis. One dog underwent MRI at 90 days post-operatively, as a routine checkup. Two dogs underwent a second MRI at 90 days post-operatively (Figure 6) to assess if there were relevant cord changes since radiographs showed the breaking of a screw and mild-to-moderate subsidence. In both cases, no relevant MRI changes or residual or new cord compressions were identified. In two cases, MRI was also repeated at 12 and 36 months and showed progression of the intramedullary damage in both cases. The damage contributed to a mild worsening of the gait only in the dog that had MRI at 36 months.

**Figure 7:**
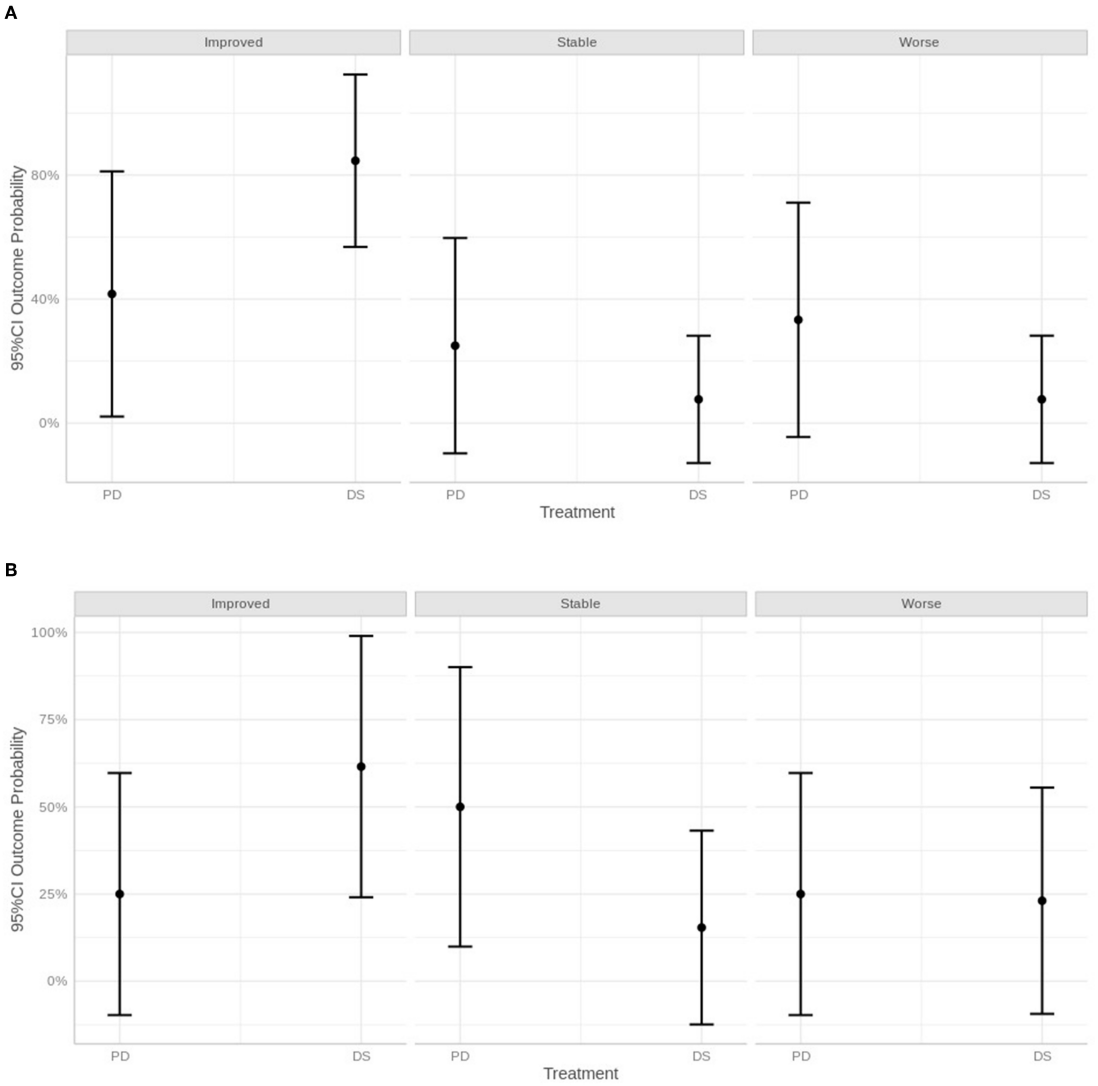
Graphical representation of neurological status within 30 days of surgery **(A)**, after 30 days and within 1 year **(B)** and over 1 year, defined as improved, stable, or worse.

## Discussion

The results of this study suggest that DA-CSM, despite being a challenging disease, could be surgically treated successfully using the PD and DS techniques. However, DS with cage, plates, and transarticular screws seemed superior to PD, with fewer clinical and radiographic failures.

DA-CSM in Dobermann and other large breed dogs more frequently affected the C6-C7 intervertebral disc space. In 35% of our cases, the C5-C6 was also involved, with subsequent single or multiple compression and damage to the spinal cord (1–5). According to the dynamic response on myelography, computed tomography, or MRI studies, it is possible to distinguish between static and dynamic lesions or, more precisely, between traction non-responsive and traction-responsive cord compressions (1, 5, 8–15, 20–22).

The preferred treatment for dynamic DA-CSM lesions is still controversial. Medical management generally results only in transient clinical improvement, and progression to severe tetraparesis is common (1, 11, 23–26). A study comparing conservative and surgical treatment strategies found that a beneficial outcome was associated with non-surgical treatment in 54% of dogs and with surgical therapy in 81% of dogs. However, the difference was not statistically significant, probably because of the low patient number (26). Overall, there is a tendency to perceive surgical treatment as superior relative to conservative options, especially in the medium-long term. The same is observed in human studies (57). A variety of surgical techniques have been proposed for the treatment of DA-CSM in dogs, with success rates between 70 and 90% (27–45). The purpose of surgical intervention should be to improve neurological deficits or, in more severe cases, to stop or slow down progression, by relieving the spinal cord compression and stabilizing the cervical vertebrae, anytime a dynamic component is suspected (1, 10). Various types of spinal decompression and vertebral stabilization techniques have also been reported for the treatment of cervical disc pathology in humans. The use of intervertebral body cages with or without adjuvant locking plates to achieve interbody arthrodesis rapidly gained acceptance in humans. More recently, the technique became popular for dogs (32–45, 57–62). Based on our previous experience of more than 30 cases, the use of the intervertebral cage alone or in combination with ventral plating was often insufficient to maintain the required intervertebral body distraction with a high incidence of subsidence, despite relatively good clinical outcomes. Good improvement and frequent subsidence (4/7 cases) have also been reported elsewhere (37). We therefore adjusted our surgical technique of DS by combining the intervertebral spacer with two ventral locking plates and two dorsal transarticular screws, similar to a previously described procedure (40). In contrast to vertebral distraction stabilization, it has been suggested that normal vertebral motion should be preserved or restored. For this purpose, a prosthetic disc to provide vertebral distraction and neural decompression was designed (41–44, 57, 59, 63). We decided to compare these two very different surgical techniques by evaluating the clinical and radiographic outcomes at different times after surgery. Our results showed that the prosthetic disc technique was more prone to failure, especially soon after surgery. Although the surgery led to neurological improvement in most cases, regardless of the surgical technique, many of the dogs in the PD group deteriorated sooner than dogs in the DS group. The most critical period in PD-treated cases seemed to be the 1st month after the operation, mostly due to moderate-severe subsidence associated with worsening of cord compression, as well as disc extrusion in two cases. In some cases, a second surgery was necessary to relieve spinal compression and counteract the effects of subsidence. We hypothesized that the discs extruded due to the collapse of the intervertebral disc space with residual disc material entering the vertebral canal. When selecting a surgical technique for the management of patients with DA-CSM, the success rate and the potential risks of complications such as implant failure and subsidence should be considered (28–45, 49). Subsidence is commonly associated with many surgical techniques used to treat DA-CSM. In humans, this has been defined as the sinking of a body with a higher elasticity modulus (e.g., graft, cage, and spacer) in a body characterized by a lower elasticity modulus (e.g., vertebral body), resulting in 3D changes in the spinal geometry and eventually in a partial or total failure of the vertebral distraction-stabilization (49, 60, 62). Even in human neurosurgery, there is no consensus regarding its role in causing complications after cervical fixation-fusion surgeries. However, subsidence seems to predispose to implant failure. Subsidence may cause clinical deterioration partly due to implant failure, and also partly because the actual loss of distraction can contribute to cord compression relapse due to the remaining soft tissues at the epidural level, including the annulus fibrosus and ligaments, and also, both direct and indirect vascular cord damage, followed by abnormal vertebral motions. Many studies have tried to identify surgical techniques that minimize or eliminate subsidence. Available data showed that subsidence is not only a radiographic failure but also is often associated with clinical deterioration, especially moderate or severe subsidence, and when it occurs early in the post-operative period. Our hypothesis that subsidence is more commonly observed with the PD than the DS technique was confirmed. We hypothesized that a potential cause for the high incidence of subsidence in the PD group is the small size of the artificial disc when compared to the size of the intervertebral disc space. Based on the results of previous studies, we hypothesized that the loading surface of the prosthetic disc is too small compared to those of the vertebral endplates, which eventually tend to incorporate the disc itself (15, 63). Moreover, the stiff nature is not capable of adsorbing the vertebral movements of the caudal cervical region without sinking into the vertebral endplates themselves. As a direct consequence of severe subsidence, the caudal cervical vertebrae lose distraction and fuse, either partially or totally, and motion cannot be restored.

The most common cause of deterioration in dogs in the DS group was discospondylitis, which developed in three cases. One case worsened due to progressive intramedullary damage as identified in the 3-year post-operative MRI. Except for one dog where discospondylitis developed 45 days after surgery, and in the other two dogs, it developed relatively late, at 5 and 10 months, respectively. Surprisingly, the infected spaces were close to the treated ones but never those that had been operated on, even if we should admit that the presence of the implant could have masked an underlying minor infection. In the first case, discospondylitis occurred soon after surgery, and thus, is likely due to the surgical procedure. On the contrary, in the other two dogs, we did not identify a relevant correlation. Surgery may directly alter normal motion and the blood supply, and predispose bacterial infection, but this is possible in any spine surgery, and not necessarily related to the synthetic material *per se* (54, 64). Indeed, all dogs improved after a relatively short course of antibiotics, without the need to remove the implants, and none showed signs of relapse. Subsidence was rarer and milder in the DS group than in the PD group. Importantly, none of the DS cases developed severe subsidence, which seems to be more often related to early and severe neurological deterioration. Repeat MRI of the dogs in the DS group with subsidence did not show new or relapsed cord compression, possibly because of the mild degree of vertebral collapse. The most common cause of subsidence in these cases was the breaking of a screw, as observed in three dogs. Breaking of the screws, even if rare, could be caused when the screw size is too small, and/or by an anomalous angulation of the screws, possibly in association with excessive movement or exercise, especially in the 1st weeks after surgery. Another possible explanation may be the weakening of the adjacent vertebral endplates, which are usually drilled to a greater extent for the insertion of the cage in the DS technique, compared to PD implantation.

A major disadvantage of the DS technique described here is the long operation time, particularly if two adjacent sites must be stabilized. However, this time may be reduced with experience. We felt that the meantime reported in this case series was negatively influenced by the long duration in the first cases that underwent this surgical technique. Both PD and DS techniques had very low to no intraoperative complication rates and short hospital stays. Therefore, both procedures are safe and may be used to treat DA-CSM in dogs. A longer learning curve is required with the DS surgery, whereas PD implantation is relatively simple.

The main limitation of this study is the small number of cases that affected the statistical power. There are many controversial aspects in the field of DA-CSM, both diagnostic (e.g., performing or not performing MRI after traction) and therapeutic (e.g., decompressing and stabilizing or creating-restoring a new functional joint), which can inevitably affect some results of this work. Another limitation of this study relates to the inclusion criterion according to which patients treated with PD had to have complete resolution of spinal cord compression after traction. Even if this was decided to reduce the chances of a worse outcome, by doing so, we could have limited the failure rates reported in this paper. Our results suggest that DS is superior to PD implantation in veterinary neurosurgery. The DS technique provided better short- and long-term clinical and radiographic outcomes in patients with DA-CSM. However, our study was limited by the small sample size. As such, we could not support this conclusion statistically. Future studies with larger sample sizes to verify our preliminary results are warranted.

## Data Availability Statement

The original contributions presented in the study are included in the article/supplementary material, further inquiries can be directed to the correspondingauthor.

## Ethics Statement

The animal study was reviewed and approved by Internal Institutional Diagnostica Piccoli Animali Ethics Committee. Written informed consent for participation was not obtained from the owners because animals were part of our daily work flow, before becoming part of this study.

## Author Contributions

CF and NG gave an essential contribution in designing the work and developing it, by analyzing the MRIs, operating the dogs, collecting the data, and actively contributed to the writing of the article. VT helped in designing the statistics for the study, analyzed all the data collected, and did all the statistic test necessary for defining the data's relevant value. All authors contributed to the article and approved the submitted version.

## Conflict of Interest

The authors declare that the research was conducted in the absence of any commercial or financial relationships that could be construed as a potential conflict of interest.

## Publisher's Note

All claims expressed in this article are solely those of the authors and do not necessarily represent those of their affiliated organizations, or those of the publisher, the editors and the reviewers. Any product that may be evaluated in this article, or claim that may be made by its manufacturer, is not guaranteed or endorsed by the publisher.
